# Predictors and prognostic impact of spontaneous portal vein thrombosis recanalization in cirrhosis: An individual patient data meta-analysis

**DOI:** 10.1097/HC9.0000000000001070

**Published:** 2026-11-11

**Authors:** Antonio Guerrero, Laura del Campo, Angelo Luca, Pierre-Emmanuel Rautou, Sylvie Chevret, Leonard Naymagon, Fabio Piscaglia, Horia Ștefănescu, Thomas Reiberger, Guohong Han, Francesco Violi, Carlos-Noronha Ferreira, Luis Téllez, Bernhard Scheiner, Stefania Basili, Javier Zamora, Agustín Albillos

**Affiliations:** 1 Servicio de Gastroenterología y Hepatología, Hospital Universitario Ramón y Cajal, Universidad de Alcalá, Instituto Ramón y Cajal de Investigación Sanitaria (IRYCIS), Madrid, España; 2 Centro de Investigación Biomédica en Red de Enfermedades Hepáticas y Digestivas (CIBEREHD), Instituto Salud Carlos III, Madrid, España; 3 Unidad de Bioestadística Clínica, Hospital Universitario Ramón y Cajal, Instituto Ramón y Cajal de Investigación Sanitaria (IRYCIS), Consorcio de Investigación Biomédica en Red de Epidemiología y Salud Pública (CIBERESP), Madrid, España; 4 Department of Diagnostic and Therapeutic Services Associate Professor of Radiology – University of Pittsburgh. IRCCS-ISMETT, Palermo, Italy; 5 IRCCS-ISMETT, Palermo, Italy; 6 Université Paris-Cité, Inserm, Centre de recherche sur l’inflammation, UMR 1149, Paris, France; 7 AP-HP, Hôpital Beaujon, Service d’Hépatologie, DMU DIGEST, Centre de Référence des Maladies Vasculaires du Foie, FILFOIE, ERN RARE-LIVER, Clichy, France; 8 Institut de Recherche Saint-Louis, équipe ECSTRRA, Université Paris Cité, Paris, France; 9 Tisch Cancer Institute, Icahn School of Medicine at Mount Sinai, New York, New York, USA; 10 Division of Internal Medicine, Hepatobiliary and Immunoallergic Diseases, IRCCS Azienda Ospedaliero-Universitaria di Bologna, Bologna, Italy; 11 Department of Medical and Surgical Sciences, University of Bologna, Bologna, Italy; 12 Gastroenterology Department, Regional Institute of Gastroenterology and Hepatology O. Fodor, Cluj-Napoca, Romania; 13 Division of Gastroenterology and Hepatology, Department of Medicine III, Medical University of Vienna, Rare Liver Disease (RALID) Center of the European Reference Network for Rare Hepatological Diseases (ERN RARE-LIVER), Vienna, Austria; 14 Department of Liver Diseases and Interventional Radiology, Digestive Diseases Hospital, Xi’an International Medical Center Hospital, Northwestern University, Xi’an, China; 15 Department of Liver Diseases and Digestive Interventional Radiology, National Clinical Research Center for Digestive Diseases, Xijing Hospital of Digestive Diseases, Fourth Military Medical University, Xi’an, China; 16 Department of Internal Medicine, Anestesiology and Cardiovascular Sciences, Sapienza University of Rome, Rome, Italy; 17 Serviço de Gastrenterologia e Hepatologia, Hospital de Santa Maria, Centro Hospitalar Universitário Lisboa Norte, Clínica Universitária de Gastrenterologia, Faculdade de Medicina, Lisbon, Portugal

**Keywords:** competing risks, liver transplantation, survival

## Abstract

**Background:**

Portal vein thrombosis (PVT) in cirrhosis has a quite unpredictable natural history. Spontaneous recanalization may occur, but predictors and prognostic impact remain uncertain. We performed an individual patient data (IPD) meta-analysis to evaluate survival according to spontaneous recanalization and to identify predictors of recanalization.

**Methods:**

We included cohort studies of patients with cirrhosis and PVT not receiving anticoagulation and obtained de-identified IPD. All-cause mortality was analyzed using a competing-risk model (competing event: liver transplantation) adjusted for prespecified baseline covariates. Predictors of recanalization were assessed with mixed-effects ordinal logistic regression including a random study intercept.

**Results:**

Eight studies contributed 586 patients. Spontaneous recanalization occurred in 264 (47.7%). Overall recanalization was independently associated with lower mortality (aSHR 0.51; 95% CI 0.28–0.90), with the benefit driven by complete recanalization (aSHR 0.45; 95% CI 0.21–0.94). Recanalization of PVT localized in the main portal vein trunk was linked to a strong survival benefit (interaction SHR 0.39; 95% CI 0.16–0.91; *p*=0.02), whereas no benefit was observed after recanalization of isolated intrahepatic branch thrombosis. PVT recanalization was more likely to occur in Child–Pugh A (aOR 2.63; 95% CI 1.18–5.88), partial thrombosis (aOR 1.69; 95% CI 1.09–2.56), and isolated intrahepatic location (aOR 2.04; 95% CI 1.23–3.33).

**Conclusions:**

Spontaneous recanalization occurs in approximately half of patients with cirrhosis and PVT and is associated with improved survival, particularly when complete and involving the main portal vein trunk. These findings underscore its prognostic relevance and indirectly support anticoagulation to promote recanalization.

## Graphical abstract

**Figure:**
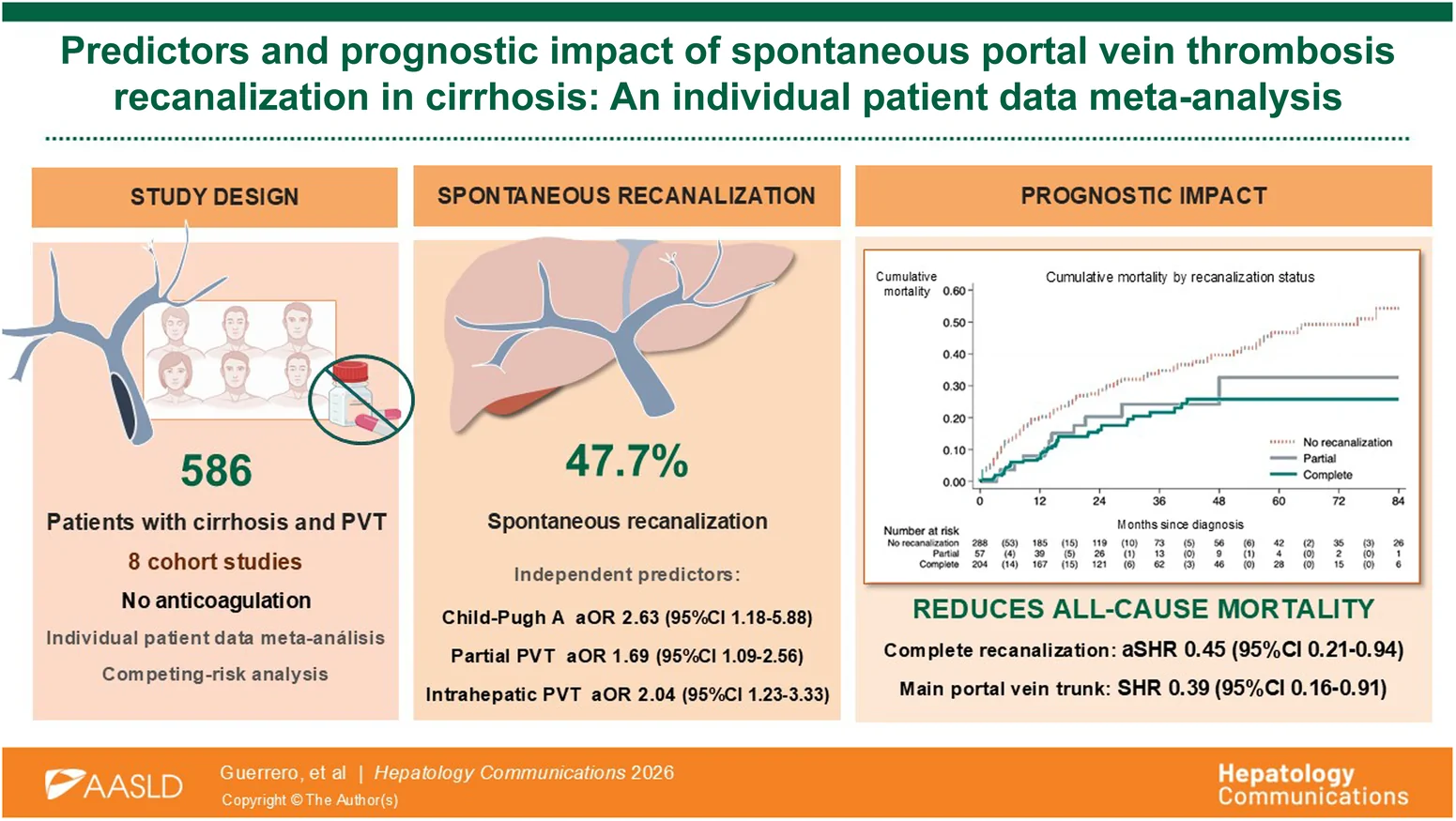

## INTRODUCTION

Portal vein thrombosis (PVT) is a common complication of cirrhosis, with prevalence increasing with disease severity and a 5-year incidence of 8.4% reported in a large prospective cohort.^1^,^2^ Its natural history is dynamic: thrombi may progress, remain stable, or regress without anticoagulation, and prospective series suggest spontaneous recanalization in around 40% of untreated patients.^3^ Retrospective cohorts and aggregate-data meta-analyses indicate that lower thrombosis burden and better preserved liver function are associated with higher rates of spontaneous resolution.^4^,^5^ However, heterogeneous imaging schedules and limited confounder adjustment leave the true frequency and predictors of spontaneous recanalization incompletely defined.

The prognostic relevance of PVT is also uncertain. Beyond acute variceal bleeding, where PVT has been associated with higher early treatment failure and short-term mortality,^6^ most observational studies have not shown an independent impact of partial or complete PVT on long-term decompensation or survival.^4^,^7^ In contrast, in liver transplantation, extensive PVT increases surgical complexity and is linked to poorer post-transplant outcomes.^8^ Despite this uncertainty on prognosis, anticoagulation is increasingly used to promote portal vein recanalization, supported by higher recanalization rates compared with no treatment.^9^,^10^,^11^,^12^,^13^,^14^ However, outside the transplantation setting, evidence directly linking recanalization to improved clinical outcomes remains limited. Small, largely uncontrolled cohorts suggest fewer portal hypertension-related events and longer transplantation-free survival among patients who recanalize under vitamin K antagonists.^15^ Moreover, an individual patient data (IPD) meta-analysis reported fewer liver-related events and improved overall survival with anticoagulation irrespective of full recanalization.^16^ As any potential benefit must be weighed against bleeding risk, particularly gastrointestinal, clarifying the prognostic meaning of spontaneous recanalization and identifying patients most likely to recanalize without intervention remain unmet needs.

To address these gaps, we performed an IPD meta-analysis to evaluate the association between spontaneous recanalization and overall survival and to quantify and identify predictors of spontaneous recanalization in patients with cirrhosis and PVT.

## METHODS

### Study design and protocol registration

This systematic review and individual patient data (IPD) meta-analysis was undertaken to characterize the natural history of PVT in cirrhotic patients not exposed to anticoagulation. We aimed to include studies that compared anticoagulation versus no treatment and cohorts consisting exclusively of untreated patients. The protocol was prospectively registered in PROSPERO (CRD42020140026) and follows PRISMA guidelines.^17^ All research was conducted in accordance with both the Declarations of Helsinki and Istanbul. The present individual participant data meta-analysis was approved by the Research Ethics Committee for Medicinal Products (CEIm) of Hospital Universitario Ramón y Cajal, Madrid, Spain (reference 030/26), which waived the requirement for additional informed consent for this secondary analysis of pseudonymised data.

### Search strategy

An experienced medical librarian (Noelia Álvarez﻿) developed and executed the search strategy (Supplemental Table S1, https://links.lww.com/HC9/C494). We queried MEDLINE, Embase, and the Cochrane Central Register of Controlled Trials from inception through March 2023. Search terms included “portal vein,” “venous thrombosis,” “portal thrombosis,” “liver cirrhosis,” “anticoagulation,” “untreated,” and “natural history.” In addition, we hand-searched reference lists of relevant articles and personal bibliographies to identify any additional eligible studies.

### Study selection

Two independent reviewers (Antonio Guerrero and Luis Téllez) screened titles and abstracts for eligibility. We included randomized controlled trials or cohort studies of adult cirrhotic patients diagnosed with PVT who had not received vitamin K antagonists (VKAs) or low-molecular-weight heparin (LMWH) at baseline. We further required studies to report on spontaneous recanalization, predictive factors thereof, or the impact of portal vein recanalization on clinical outcomes. Languages were limited to English, Spanish, French, Italian, and German. Exclusion criteria comprised: (1) PVT in non-cirrhotic patients; (2) post-transplant or postoperative PVT; (3) interventions involving transjugular intrahepatic portosystemic shunt, thrombolysis, or direct oral anticoagulants (DOACs). Discrepancies in study selection were resolved by consensus or by consulting a third reviewer (Agustín Albillos or Javier Zamora).

### Data acquisition and extraction

Corresponding authors of eligible publications were contacted to contribute de-identified IPD. Two reviewers (Antonio Guerrero and Luis Téllez) independently extracted data from published reports and checked original datasets for completeness and consistency, reconciling any discrepancies through follow-up correspondence with study investigators. We collected patient-level variables including demographics (age, sex), cirrhosis etiology, laboratory values [creatinine, bilirubin, albumin, prothrombin time/international normalized ratio (INR), platelet count], Child–Pugh class and score, Model for End-Stage Liver Disease (MELD) score, cirrhosis complications (ascites, encephalopathy, portal-hypertensive bleeding), variceal bleeding prophylaxis (eg, beta-blocker use), PVT characteristics (severity, location), follow-up duration, and dates of liver transplantation or death. Patients lacking sufficient data to ascertain outcomes were excluded from the analysis.

### Quality assessment

Risk of bias for each included study was independently appraised by two authors (Antonio Guerrero and Laura del Campo) using the Quality in Prognostic Studies (QUIPS) tool^18^ (Supplemental Table S2.1, S2.2, https://links.lww.com/HC9/C494).

### Definitions and outcome measures

We used the definition of PVT referred to by the investigators of the included studies. PVT was defined as the absence or reduction in flow in any segment of the splanchnic venous system caused by intraluminal thrombosis, as documented by Doppler ultrasound, contrast-enhanced CT, or MRI. Severity was categorized as “partial” or “complete” based on each study’s criteria. Thrombosis location was grouped into: (1) portal vein trunk (with or without branch involvement), (2) intrahepatic portal vein branches alone, or (3) mesenteric veins.

The primary end point was all-cause mortality, which was defined as death from any cause during follow-up. The secondary end point was spontaneous portal vein recanalization, defined as any documented reduction in thrombosis length or thickness (partial recanalization) or complete disappearance of thrombosis (complete recanalization).

### Statistical analysis

The differences in baseline characteristics between non-spontaneous and spontaneous recanalization groups were evaluated using a chi-square test, Student *t* test, or Mann–Whitney *U* test according to normality of data distribution. The proportional hazards assumption was evaluated using both graphical and statistical approaches.^19^ First, log–log survival curves were inspected. Second, a formal test based on Schoenfeld residuals was performed.^20^. To assess the impact of recanalization on all-cause mortality, we performed a one-stage IPD meta-analysis using a Fine and Gray competing risks model, treating liver transplantation as a competing event. This model accommodates the correlation within studies using a sandwich variance estimator. Time to death or transplantation was calculated as the difference between the date of PVT and the date of death or transplantation, respectively. The model was adjusted a priori for the following covariates, selected on clinical grounds to avoid overfitting: age, cirrhosis etiology (alcohol, viral, or other), cirrhosis severity (MELD, Child–Pugh class A vs. B/C), PVT severity (partial vs. complete) and location (mesenteric, intrahepatic, or portal trunk), platelet count, presence of esophageal varices, and beta-blocker use for variceal bleeding prophylaxis. Between-study heterogeneity was evaluated by fitting a proportional hazards model with a shared gamma frailty parameter and estimating the variance component. We plotted cumulative incidence curves and estimated 1-year, 2-year, and 6-year survival probabilities for recanalization groups.

Given that portal vein recanalization can be classified on an ordinal scale (ie, complete, partial, or absent), we used an ordinal logistic regression model with a random intercept for each study to identify independent predictors of increasing degree of spontaneous recanalization. This approach exploits the intrinsic ordered nature of the outcome, allowing estimation of proportional odds ratios that reflect the likelihood of achieving a more favorable degree of recanalization (ie, transitioning from no recanalization to partial, or from partial to complete), while preserving the full informational gradient of the response variable. All statistical tests were two-tailed, and significance was set at *p*<0.05. Stata version 18 was used for all analyses.

## RESULTS

### Study selection

Our comprehensive search yielded 223 references (Figure 1). After duplicate removal, 183 articles underwent title and abstract screening, whereas 146 were excluded for not meeting the inclusion criteria. The full texts of the remaining 37 studies were retrieved, and 23 were subsequently excluded based on predefined criteria. We contacted the corresponding authors of the 19 eligible articles, and 9 did not respond despite multiple follow-up attempts, and 2 declined to share data. Five of the 19 eligible articles corresponded to cohorts already included in the IMPORTAL study.^16^ Ultimately, 8 studies, encompassing 586 non-anticoagulated patients with cirrhosis and PVT, were included in the IPD meta-analysis.

**FIGURE 1:**
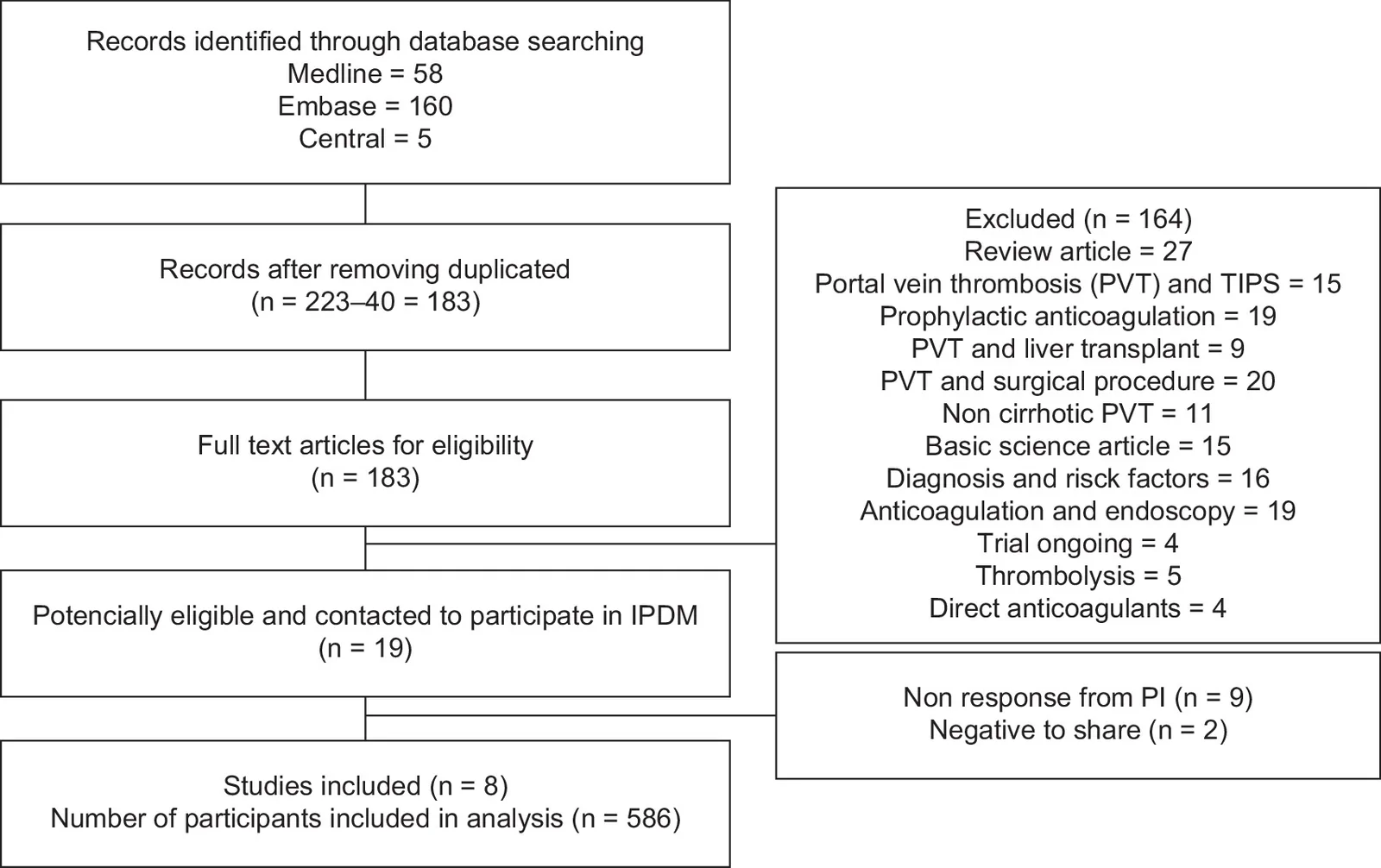
PRISMA flow diagram showing the study identification, screening, eligibility, and inclusion process for the systematic review. Abbreviation: IPDM, individual patient data meta-analysis; PI, principal investigator; PRISMA, Preferred Reporting Items for Systematic Reviews and Meta-Analyses; PVT, portal vein thrombosis.

### Study characteristics

Table 1 summarizes key features of the 8 included cohorts. All analyses were retrospective, although 3 studies (Nery et al,^3^ Violi et al,^12^ Noronha Ferreira et al^9^) originally enrolled patients prospectively. Each study enrolled adult cirrhotic patients diagnosed with PVT and explicitly excluded individuals with tumor-related PVT or extrahepatic malignancies. The date of PVT diagnosis was uniformly defined as the first image demonstration of thrombosis. In 5 studies (Violi et al,^12^ Noronha Ferreira et al,^9^ Pettinari et al,^10^ Chen et al,^11^ Scheiner et al,^13^ Naymagon et al^14^), cohorts were divided into anticoagulated and non-anticoagulated arms, and for our analysis, we extracted IPD exclusively from the non-anticoagulated groups. In the study of Nery et al,^3^ all but 6 patients included were not treated with anticoagulation, and the study of Luca et al^7^ enrolled solely non-anticoagulated patients.

**TABLE 1:** Characteristics of the 8 studies included in the meta-analysis

Study, year	Type of study	Number of patients	Inclusion criteria	Exclusion criteria	Definition of thrombosis severity	Definition of recanalization	Thrombosis severity	Thrombosis location	Imaging	Duration of follow-up (median, months)
Luca et al, 2012^7^	Retrospective	Total 42 No AC: 42	Cirrhosis and PVT	Tumoral PVT Complete PVT TIPS	Complete: No residual lumen Partial: Any residual lumen	Complete: No residual thrombosis Partial: At least >10% improvement in lumen occlusion	Partial: 100%	Main trunk of portal vein: 100%	Diagnosis: CT Follow-up: CT	27
Nery et al, 2015^3^	Prospective	Total: 118 No AC: 118	Cirrhosis and PVT	Tumoral PVT Extrahepatic malignancy Extrahepatic disease life expectancy <1 year	Complete: Absence of blood flow Partial: Any blood flow detected	ND	Compete: 18% Partial: 82%	Main trunk of portal vein: 74.4% Isolated intrahepatic vein: 25.5%	Diagnosis: DUS and CT/MRI Follow-up: DUS	47
Violi et al, 2016^12^	Prospective	Total: 126 AC: 45 No AC: 81	Cirrhosis and PVT	Tumoral PVT Extrahepatic malignancy	Complete: Absence of blood flow Partial: any blood flow detected	ND	Complete: 15% Partial: 85%	Main trunk of portal vein: 76% Isolated intrahepatic branches: 23% Isolated mesenteric vein: 1.5%	Diagnosis: DUS Follow-up: DUS	24
Chen et al, 2016^11^	Retrospective	Total: 66 AC: 30 No AC: 36	Cirrhosis and PVT	Intrahepatic or extrahepatic malignancy TIPS Splanchnic vein thrombosis without PVT Anticoagulation pre-PVT	Complete: >75% lumen occupancy Partial: <75% lumen occupancy	Complete: No residual thrombosis Partial: Any improvement in thickness or length	Complete: 65% Partial: 35%	Main trunk of portal vein: 100%	Diagnosis: CT Follow-up: CT	11.7
Pettinari et al, 2019^10^	Retrospective	Total: 182 AC: 81 No AC: 101	Cirrhosis and PVT	Malignancy Anticoagulation pre-PVT	Complete: >90% lumen occupancy Partial: any blood flow detected	Complete: No residual thrombosis Partial: At least >50% improvement in thickness or length	Complete: 15% Partial: 85%	Main trunk of portal vein: 73% Isolated intrahepatic branches: 22% Isolated mesenteric vein: 5%	Diagnosis: DUS Follow-up: DUS at 3, 6, and 12±12 mo	19
Scheiner et al, 2018^13^	Retrospective	Total: 51 AC: 12 No AC: 39	Cirrhosis and PVT	Tumoral PVT	ND	ND	Complete: 40% Partial: 60%	Main trunk portal vein: 100%	ND	44
Noronha Ferreira et al, 2019^9^	Retrospective	Total: 80 AC: 37 No AC: 43	Cirrhosis and PVT	Tumoral PVT Extrahepatic malignancy TIPS Splanchnic vein thrombosis without PVT	Complete: Absence of blood flow Partial: Any blood flow detected	Complete: No residual thrombosis Partial: At least >25% improvement in thickness or length	Complete: 45% Partial: 55%	Main trunk of portal vein: 85% Isolated intrahepatic branches: 15%	Diagnosis: DUS and CT/MRI Follow-up: DUS and CT/MRI at 6 mo, then DUS every 6 mo	25
Naymagon et al, 2021^14^	Retrospective	Total: 214 AC: 86 No AC: 128	Cirrhosis and PVT	Tumoral PVT Extrahepatic malignancy TIPS/thrombectomy Splanchnic vein thrombosis without PVT Cavernous transformation	ND	Complete: No residual thrombosis Partial: Resolution of fully occlusive Conversion from fully to non-occlusive	Complete: 24% Partial: 76%	Main trunk of portal vein: 78.8% Isolated intrahepatic branches: 18% Isolated mesenteric vein: 3.2%	Diagnosis: DUS and CT/MRI Follow-up: CT	27

Abbreviations: AC, anticoagulation; CT, computed tomography; DUS, Doppler ultrasound; MRI, magnetic resonance imaging; ND, not defined; PVT, portal vein thrombosis; TIPS, transjugular intrahepatic portosystemic shunt.

Definitions of PVT severity varied: 4 studies defined complete thrombosis as the absence of any residual flow channel in the portal trunk,^3^,^7^,^9^,^12^ whereas the other 2 applied thresholds of ≥90% and ≥75% luminal occlusion, respectively.^10^,^11^ Scheiner et al^13^ and Naymagon et al^14^ offered no explicit criteria for complete versus partial PVT. Recanalization definitions were heterogeneous as well. Four cohorts considered partial recanalization as a 10%–50% reduction in thrombosis length or thickness and complete recanalization as the absence of visible thrombosis.^7^,^9^,^10^,^11^ Naymagon et al^14^ defined partial recanalization more broadly as conversion from fully occlusive to non-occlusive PVT or any reduction of a previously occlusive thrombosis. Violi et al^12^ and Scheiner et al^13^ did not specify recanalization criteria.

Imaging protocols differed across studies: 3 primarily relied on Doppler ultrasound for both diagnosis and follow-up (Table 1), with median follow-up durations ranging from 11.7 to 47 months. Four cohorts systematically confirmed PVT by CT or MRI.^3^,^9^,^12^,^14^ Chen et al and Luca et al used contrast-enhanced CT alone, while Scheiner et al did not describe their imaging modality. Except for Luca et al, which enrolled only partial PVT, all other studies included partial and complete PVT. Six cohorts also enrolled individuals with isolated intrahepatic portal branch thrombosis (range, 15%–25% of patients). Isolated superior mesenteric vein thrombosis was uncommon (1.5%–5% of patients) and represented only in 3 studies.^10^,^12^,^14^

### Quality assessment

We assessed study quality using the QUIPS tool for both outcomes—survival and spontaneous recanalization (Supplemental Tables S2.1–S2.2, https://links.lww.com/HC9/C494). Seven of the 8 studies were rated as having an overall moderate risk of bias for both outcomes, whereas Scheiner et al^13^ was judged high risk, mainly driven by a high risk of attrition bias (unreported reasons for patient exclusion and incomplete data on missing cases) and moderate risk in prognostic factor measurement. Across studies, most domains were reassuring, with low risk of bias for study participation, confounding, and statistical analysis/reporting. For overall survival, the main concern was study attrition, rated moderate in most cohorts^3^,^7^,^9^,^10^,^11^,^12^,^14^ and high in one,^13^ which could introduce selection bias if dropouts were differential. For spontaneous recanalization, the principal limitation was outcome measurement, rated moderate in several studies^3^,^7^,^10^,^12^ and high in one,^13^ reflecting variability in imaging modality, and operational definitions of recanalization.

### Patient characteristics

Characteristics of the 586 patients of the full cohort are shown in Table 2, with study-specific data in Supplemental Tables S3–S10, https://links.lww.com/HC9/C494. Most patients were male (64.6%), and cirrhosis etiologies were most often viral (46.8%) or alcohol-associated (33.8%). At PVT diagnosis, 38.5% of patients were classified as Child–Pugh A, while 61.5% were Child–Pugh B/C, with a mean (SD) MELD of 12.8 (5.1). Overall, 23.8% had complete thrombosis, and 79.4% exhibited thrombosis in the main portal trunk, whereas 18.2% had isolated intrahepatic branch involvement.

**TABLE 2:** Baseline characteristics of patients from the 8 studies included in the IPD meta-analysis at the time of portal vein thrombosis diagnosis

	N=586	Missings (%)
Age, mean (SD)	58.3 (11.6)	1 (0.2%)
Min.–max.	20–87	
Gender		1 (0.2%)
Male	378 (64.6%)	
Female	207 (35.4%)	
Etiology		1 (0.2%)
Viral	274 (46.8%)	
Alcohol	198 (33.8%)	
Others	113 (19.3%)	
Child–Pugh		
Mean (SD)	6.8 (3.3)	12 (2.1%)
Child–Pugh A	221 (38.5%)	12 (2.0%)
Child–Pugh B/C	353 (61.5%)	12 (2.0%)
MELD score		
Mean (SD)	12.8 (4.9)	40 (6.8%)
Beta-blockers	251 (45.6%)	35 (6.0%)
Varices	453 (82.1%)	34 (5.8%)
Platelets, median (P25th–P75th)	86 (56–135)	77 (13.1%)
Min.–max.	9–561	
Portal vein thrombosis severity		11 (1.9%)
Partial	438 (76.2%)	
Complete	137 (23.8%)	
Localization		9 (1.6%)
Main trunk	458 (79.4%)	
Intrahepatic	105 (18.2%)	
Mesenteric	14 (2.4%)	
Thrombosis status		33 (5.6%)
Recanalization	264 (47.7%)	
No recanalization	289 (52.3%)	
Detailed thrombosis status		80 (13.6%)
Stable	135 (23.3%)	
Partial recanalization	59 (11.3%)	
Complete recanalization	205 (41.3%)	
Progression	107 (21.6%)	
Status at last follow-up		1 (0.2%)
Alive	376 (64.3%)	
Death	162 (27.7%)	
Liver transplant	47 (8.0%)	

Abbreviations: IPD, individual patient data; MELD, Model for End-Stage Liver Disease; SD, standard deviation.

### All-cause mortality

During a median follow-up of 30.8 months (IQR: 18.3, 61.4 mo), 162 of 586 patients (27.7%) died. Recanalization status at last follow-up was available for 553 patients: 264 (47.7%) in the spontaneous recanalization group, compared with 289 (52.3%) in the non-recanalization group. Median survival was significantly longer among patients with spontaneous recanalization [92.8 mo; 95% CI (61.3–not estimable)] versus those without [52.7 mo; 95% CI (36.0–75.8)]. Survival probabilities at 1, 2, and 6 years for each group appear in Table 3.

**TABLE 3:** Estimated probability of transplant-free survival at prespecified time points

	Spontaneous recanalization (n=264)	No spontaneous recanalization (n=289)
Median survival time (95% CI)	92.8 (61.3–NR)	52.7 (36.0–75.8)
Survival probability, % (95% CI)		
12 months	90.4% (86.0–93.5)	77.7% (72.2–82.3)
24 months	77.8% (71.8–82.7)	65.6% (59.1–71.3)
72 months	59.1% (49.4–67.5)	42.0% (33.7–50.0)

*Note:* NR: median not reached within the observed follow-up. Failure event: death or liver transplantation (composite end point), whichever occurred first. Kaplan–Meier estimates.

Abbreviations: CI, confidence interval; NR, not reached.

The competing-risk regression (Fine–Gray) treating liver transplantation as a competing event and adjusting for age, cirrhosis etiology, MELD score, Child–Pugh class, thrombosis severity and location, beta-blocker use, platelet count, and the presence of esophageal varices showed that complete recanalization was associated with the largest reduction in all-cause mortality (aSHR=0.45; 95% CI, 0.21–0.94; *p*=0.036), whereas partial recanalization showed a non-significant trend in the same direction (aSHR=0.67; 95% CI, 0.32–1.40; *p*=0.29), suggesting a stepwise survival benefit with increasing thrombosis resolution (Figure 2 and Table 4). Consistently, when we analyzed the effect of spontaneous recanalization overall, we found that it was independently associated with a significantly lower risk of death (aSHR=0.51; 95% CI, 0.28–0.91; Supplemental Table S11, https://links.lww.com/HC9/C494).

**FIGURE 2:**
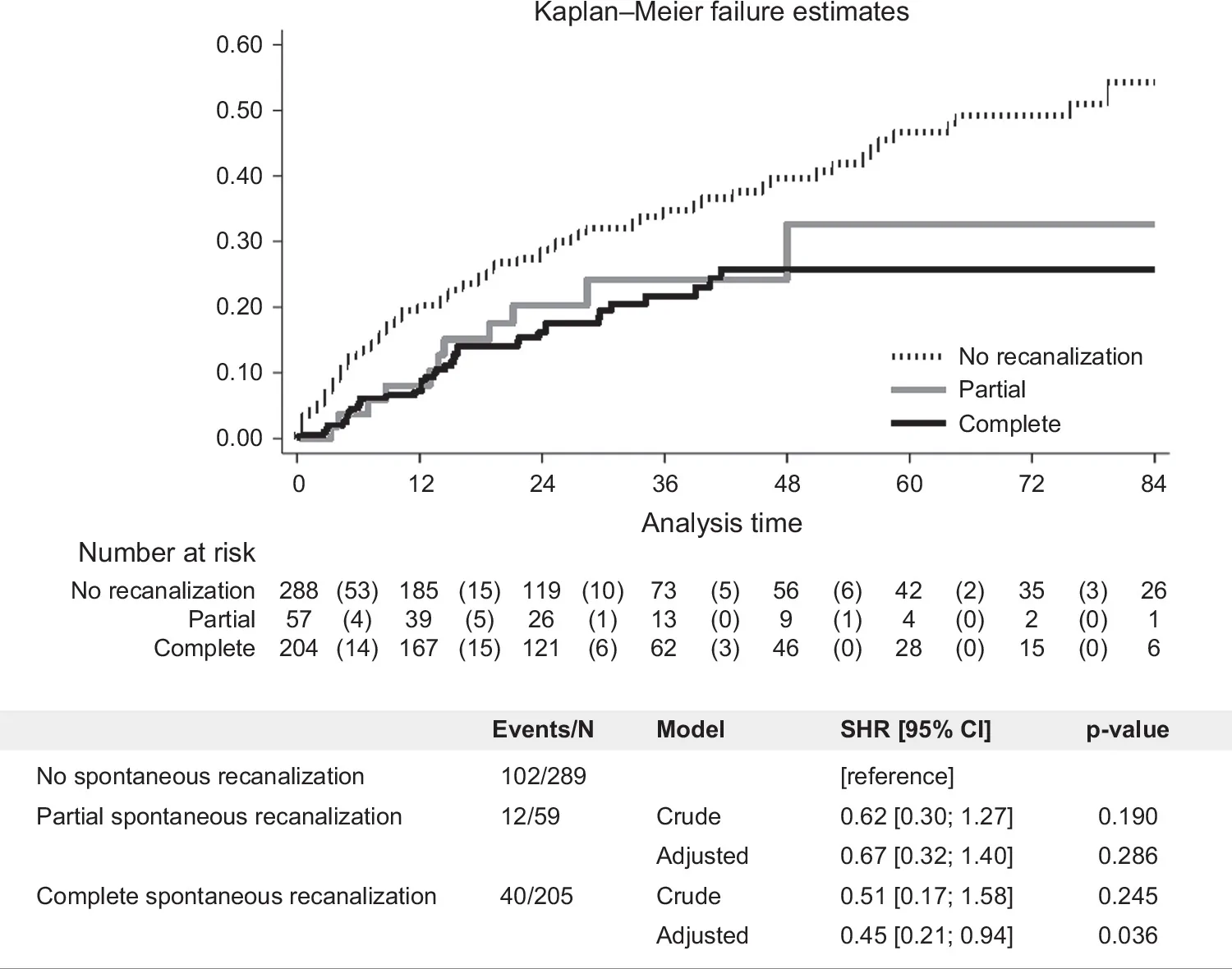
Cumulative incidence of all-cause mortality by grade of spontaneous recanalization, estimated using competing-risk analysis. Abbreviations: CI, confidence interval; SHR, sub-hazard ratio.

**TABLE 4:** Adjusted sub-hazard ratios (aSHRs) from the final multivariable competing-risk model assessing the association of complete and partial spontaneous recanalization on all-cause mortality

Variable	aSHR [95% CI]	*p*
Partial spontaneous recanalization	0.67 [0.32; 1.40]	0.286
Complete spontaneous recanalization	**0.45 [0.21; 0.94]**	**0.036**
Age (year)	**1.04 [1.03; 1.06**]	**<0.001**
MELD, points	**1.10 [1.06; 1.14]**	**<0.001**
Child–Pugh		
A	[reference]	
B/C	**2.10 [1.47; 2.96]**	**<0.001**
Beta-blockers	**0.65 [0.52; 0.80]**	**<0.001**
Etiology		
Viral	[reference]	
Alcohol	1.04 [0.51; 2.13]	0.900
Others	0.88 [0.54; 1.43]	0.621
Portal vein thrombosis severity		
Partial	[reference]	
Complete	0.88 [0.66; 1.17]	0.388
Portal vein thrombosis location		
Main trunk	[reference]	
Intrahepatic	0.95 [0.57; 1.59]	0.854
Mesenteric	0.84 [0.43; 1.65]	0.618
Platelets, 10^3^/µL	0.95 [0.60; 1.50]	0.831
Varices	0.70 [0.36; 1.36]	0.292

Bold values are Statistical significance of *P* < 0.05 value.

Log–log survival curves were approximately parallel, and Schoenfeld residuals were non-significant, supporting the proportional hazards assumption. The estimated frailty variance was small (0.23; 95% CI, 0.04–0.57), suggesting limited differences in baseline hazard across studies and only minor residual heterogeneity beyond that explained by the measured covariates. The likelihood ratio test nevertheless confirmed a modest but statistically significant between-study variability (*χ* ^2^=19.7; *p*<0.001).

To evaluate whether location and baseline severity modified the association between spontaneous recanalization and mortality, we performed a subgroup analysis. Thrombosis location significantly modified this association (*p* for interaction=0.02), with a greater reduction in mortality when thrombosis involved the main portal vein trunk (SHR 0.39; 95% CI, 0.16–0.91) (Figure 3). In contrast, no mortality benefit was observed in patients with thrombosis of an intrahepatic branch of the portal vein, irrespective of the degree of recanalization. Baseline thrombosis severity did not modify the impact of recanalization: the association with mortality was similar in patients with partial versus complete thrombosis at baseline.

**FIGURE 3:**
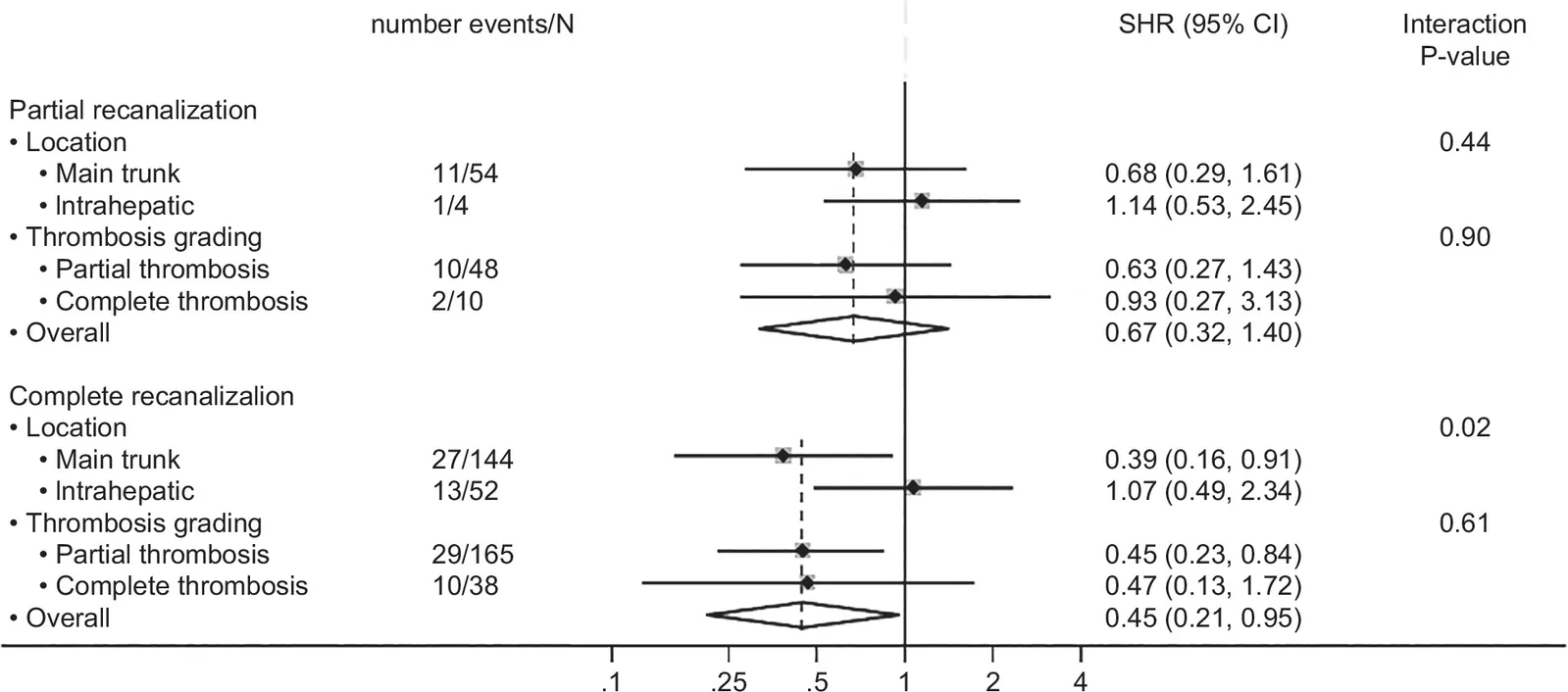
Subgroup analysis of the association between spontaneous recanalization and survival according to thrombosis severity and location. For 33 patients (5.6%), follow-up data on recanalization status were unavailable. Abbreviations: CI, confidence interval; SHR, sub-hazard ratio.

### Predictors of spontaneous recanalization

In the overall cohort, 264 of 586 patients (47.7%) achieved spontaneous recanalization during follow-up, comprising 205 complete (77.6%) and 59 partial (22.3%) recanalization (Table 2). Among the 289 patients (52.3%) without spontaneous recanalization, thrombosis status was fully documented for 242: 135 (55.7%) showed stable thrombosis, and 107 (44.2%) showed progression.

In multivariable ordinal logistic regression (Table 5), compensated cirrhosis (Child A vs. Child B/C; OR=2.63; 95% CI, 1.18–5.88; *p*=0.018), partial thrombosis (aOR=1.69; 95% CI, 1.09–2.56; *p*=0.018) and thrombosis limited to the intrahepatic branch of the portal vein (intrahepatic vs. main trunk; aOR=2.04; 95% CI, 1.23–3.33; *p*=0.005) were independently associated with higher odds of a more favorable spontaneous recanalization.

**TABLE 5:** Multivariable ordinal logistic regression analysis of baseline covariates associated with a higher probability of thrombosis recanalization

	Univariable			Multivariable		
Variable	OR	95% CI	*p*	OR	95% CI	*p*
Age (years)	1.02	[0.98, 1.07]	0.291	1.04	[0.99, 1.09]	0.084
Beta-blockers	0.44	[0.11, 1.71]	0.235	0.56	[0.27, 1.13]	0.103
**Child–Pugh A**	3.03	[0.90, 10]	0.070	**2.63**	**[1.18, 5.88]**	**0.018**
Etiology						
Alcohol	1.05	[0.52, 2.12]	0.885	0.90	[0.60, 1.36]	0.630
Other	0.74	[0.36, 1.54]	0.427	0.57	[0.23, 1.39]	0.216
**Partial thrombosis**	1.72	[0.98, 3.03]	0.060	**1.69**	**[1.09, 2.56]**	**0.018**
**Intrahepatic thrombosis**	1.78	[1.12, 2.78]	0.013	**2.04**	**[1.23, 3.33]**	**0.005**
Platelets (log)	1.05	[0.65, 1.68]	0.853	0.92	[0.64, 1.31]	0.639
MELD ≥15	0.71	[0.30, 1.69]	0.440	1.31	[0.86, 2.00]	0.205
Female	1.21	[0.78, 1.89]	0.393	1.19	[0.89, 1.59]	0.238
Varices	0.66	[0.34, 1.26]	0.207	1.11	[0.57, 2.17]	0.758

Bold values are Statistical significance of *P* < 0.05 value.

Abbreviations: CI, confidence interval; MELD, Model for End-Stage Liver Disease; OR, odds ratio.

### Sensitivity analysis

A sensitivity analysis restricted to patients with thrombosis of the main portal vein trunk showed that the protective association between recanalization and survival was maintained (complete recanalization: aSHR, 0.38; 95% CI, 0.16–0.90; *p*=0.027; any recanalization: aSHR, 0.48; 95% CI, 0.24–0.99; *p*=0.046) (Supplemental Tables S12 and S13, https://links.lww.com/HC9/C494). The main predictors of recanalization, namely Child–Pugh class and degree of thrombosis, were also preserved (Supplemental Table S14, https://links.lww.com/HC9/C494).

## DISCUSSION

This IPD meta-analysis involved a cohort of 586 patients with cirrhosis and PVT from 8 studies, including patients not receiving anticoagulation. The analysis showed that: (i) spontaneous recanalization occurred in nearly half of the patients, (ii) the likelihood of spontaneous recanalization depended on the location and severity of PVT and the severity of liver disease, and more importantly, (iii) spontaneous recanalization was associated with reduced all-cause mortality when recanalization occurred in patients with thrombosis involving the main portal vein trunk.

A key observation of our study was that spontaneous recanalization occurred in nearly 50% among patients with cirrhosis and PVT, confirming that this is a relatively common phenomenon. Among patients who recanalized, more than two-thirds achieved complete recanalization, indicating that portal flow may be fully restored without intervention in a substantial proportion of patients. Independent predictors of spontaneous recanalization were thrombosis limited to the intrahepatic portal branches rather than involving the main portal vein trunk, partial rather than complete thrombosis, and compensated cirrhosis (Child–Pugh A vs. B/C). Taken together, these results suggest that a lower thrombotic burden and preserved hepatic functional reserve favor spontaneous recanalization.

A relevant finding of our analysis was the independent association between the extent of spontaneous PVT recanalization and overall survival. Complete recanalization conferred the greatest benefit, with a 55% reduction in the risk of death. This association was demonstrated using IPD and a competing-risk model that considered liver transplantation as a competing event and adjusted for established prognostic factors, including Child–Pugh class, MELD score, and markers of portal hypertension severity. These findings, obtained in patients with PVT who were not receiving anticoagulation, may appear to contrast with our previous IPD meta-analysis of anticoagulation in cirrhosis and PVT, in which the survival benefit of anticoagulation was independent of recanalization.^16^ However, this apparent discrepancy may reflect effects of anticoagulation beyond thrombus resolution. By limiting intrahepatic thrombogenesis and fibrogenesis, anticoagulation may favorably modify the natural history of cirrhosis.^21^ Its survival benefit may therefore extend to both patients who achieve recanalization and those who do not, potentially masking the independent prognostic effect of recanalization observed in untreated patients.

Overall, interaction analyses indicated that the prognostic relevance of spontaneous recanalization depended primarily on thrombosis location rather than baseline thrombus burden. Recanalization was associated with a 61% lower risk of death in patients with thrombosis involving the main portal vein trunk, whereas no survival benefit was observed in those with isolated intrahepatic PVT. Although any degree of recanalization was associated with lower mortality, the survival benefit appeared to be magnitude-dependent, with a greater reduction in mortality following complete rather than partial recanalization. These findings suggest that a more extensive restoration of flow through the main portal vein confers a better prognosis. This is consistent with the Poiseuille law, whereby even small increases in vessel diameter may markedly reduce vascular resistance and increase blood flow. Of note, beta-blocker use showed a trend toward less frequent recanalization but was associated with improved survival, suggesting that its overall benefit may outweigh concerns related to reduced portal flow in patients with PVT.

The main strength of our study lies in the use of IPD, which enabled time-to-event analyses while accounting for competing risks such as liver transplantation. In addition, the large cohort size and number of observed events allowed robust adjustment for clinically relevant confounders, avoiding the need for imputation or data-driven variable selection approaches, which have been criticized for increasing the risk of overfitting. Instead, we applied a clinically informed approach to confounder selection.

Our study also has several limitations. First, the included studies differed in PVT severity, location, and definitions of recanalization, introducing heterogeneity. Although between-study variability appeared limited, it remained statistically detectable. The use of IPD, multivariable adjustment, and a shared-frailty approach helps mitigate bias arising from these differences; however, residual confounding cannot be fully excluded. Second, quality appraisal indicated an overall moderate risk of bias. For the survival analysis, the main potential source of bias was attrition. However, its impact was likely limited by the objective nature of all-cause mortality and the availability of MELD and Child–Pugh scores for adjustment in most patients. The recanalization findings also warrant caution when extrapolating across settings, as variability in imaging practices, follow-up intervals, and operational definitions may have led to outcome misclassification and reduced comparability across cohorts. Third, generalizability may be limited by the high proportion of patients with viral liver disease, which differs from contemporary cohorts with a greater prevalence of MASLD-related cirrhosis. Fourth, most cohorts predated current international guidance, widespread use of direct oral anticoagulants, and standardized surveillance strategies, which may limit applicability to contemporary practice.

Our finding that spontaneous recanalization of thrombosis involving the main portal vein trunk was associated with improved survival indirectly supports the Baveno VII recommendations,^22^ reinforced in Baveno VIII (submitted for publication), to anticoagulate patients with cirrhosis and recent PVT to prevent thrombus extension and promote recanalization. This interpretation should, however, be tempered by the fact that the survival benefit was observed in an untreated cohort. Evidence that anticoagulation may improve outcomes beyond thrombus resolution further supports its potential to modify the natural history of cirrhosis. Therefore, anticoagulation should generally be indicated in patients with thrombosis involving the main portal vein trunk and no contraindications. An individualized observational strategy may nevertheless be reasonable in non-transplant candidates with compensated cirrhosis and isolated intrahepatic PVT.

## Supplementary Material

## References

[1] Turon F Driever EG Baiges A Cerda E García-Criado Á Gilabert R. Predicting portal thrombosis in cirrhosis: A prospective study of clinical, ultrasonographic and hemostatic factors. J Hepatol. 2021;75:1367–1376. 10.1016/j.jhep.2021.07.020 34333101

[2] Pan J Wang L Gao F An Y Yin Y Guo X. Epidemiology of portal vein thrombosis in liver cirrhosis: A systematic review and meta-analysis. Eur J Intern Med. 2022;104:21–32. 10.1016/j.ejim.2022.05.032 35688747

[3] Nery F Chevret S Condat B de Raucourt E Boudaoud L Rautou PE. Causes and consequences of portal vein thrombosis in 1,243 patients with cirrhosis: Results of a longitudinal study. Hepatology. 2015;61:660–667. 10.1002/hep.27546 25284616

[4] Maruyama H Okugawa H Takahashi M Yokosuka O. De novo portal vein thrombosis in virus-related cirrhosis: Predictive factors and long-term outcomes. Am J Gastroenterol. 2013;108:568–574. 10.1038/ajg.2012.452 23381015

[5] Qi X Guo X Yoshida EM Méndez-Sánchez N De Stefano V Tacke F. Transient portal vein thrombosis in liver cirrhosis. BMC Med. 2018;16:83. 10.1186/s12916-018-1069-8 PMC5989335 29871683

[6] D’Amico G De Franchis R Cooperative Study Group. Upper digestive bleeding in cirrhosis. Post-therapeutic outcome and prognostic indicators. Hepatology. 2003;38:599–612. 10.1053/jhep.2003.50385 12939586

[7] Luca A Caruso S Milazzo M Marrone G Mamone G Crino F. Natural course of extrahepatic nonmalignant partial portal vein thrombosis in patients with cirrhosis. Radiology. 2012;265:124–132. 10.1148/radiol.12112236 22891357

[8] Zanetto A Rodriguez-Kastro KI Germani G Ferrarese A Cillo U Burra P. Mortality in liver transplant recipients with portal vein thrombosis—An updated meta-analysis. Transpl Int. 2018;31:1318–1329. 10.1111/tri.13353 30230053

[9] Noronha Ferreira C Reis D Cortez-Pinto H Tato Marinho R Gonçalves A Palma S. Anticoagulation in cirrhosis and portal vein thrombosis is safe and improves prognosis in advanced cirrhosis. Dig Dis Sci. 2019;64:2671–2683. 10.1007/s10620-019-05572-z 30852769

[10] Pettinari I Vukotic R Stefanescu H Pecorelli A Morelli M Grigoras C. Clinical impact and safety of anticoagulants for portal vein thrombosis in cirrhosis. Am J Gastroenterol. 2019;114:258–266. 10.1038/s41395-018-0421-0 30538290

[11] Chen H Liu L Qi X He C Wu F Fan D. Efficacy and safety of anticoagulation in more advanced portal vein thrombosis in patients with liver cirrhosis. Eur J Gastroenterol Hepatol. 2016;28:82–89. 10.1097/MEG.0000000000000482 26513611

[12] Violi F Corazza GR Caldwell SH Perticone F Gatta A Angelico M.; PRO-LIVER Collaborators. Portal vein thrombosis relevance on liver cirrhosis: Italian Venous Thrombotic Events Registry. Intern Emerg Med. 2016;11:1059–1066. 10.1007/s11739-016-1416-8 27026379

[13] Scheiner B Stammet PR Pokorny S Bucsics T Schwabl P Brichta A. Anticoagulation in non-malignant portal vein thrombosis is safe and improves hepatic function. Wien Klin Wochenschr. 2018;130:446–455. 10.1007/s00508-018-1351-y PMC6061656 29916054

[14] Naymagon L Tremblay D Zubizarreta N Moshier E Mascarenhas J Schiano T. Safety, efficacy, and long-term outcomes of anticoagulation in cirrhotic portal vein thrombosis. Dig Dis Sci. 2021;66:3619–3629. 10.1007/s10620-020-06695-4 33151401

[15] La Mura V Braham S Tosetti G Branchi F Bitto N Moia M. Harmful and beneficial effects of anticoagulants in patients with cirrhosis and portal vein thrombosis. Clin Gastroenterol Hepatol. 2018;16:1146–1152. 10.1016/j.cgh.2017.10.016 29066371

[16] Guerrero A Campo LD Piscaglia F Scheiner B Han G Violi F.; Baveno Cooperation: an EASL consortium. Anticoagulation improves survival in patients with cirrhosis and portal vein thrombosis: The IMPORTAL competing-risk meta-analysis. J Hepatol. 2023;79:69–78. 10.1016/j.jhep.2023.02.023 36858157

[17] Stewart LA Clarke M Rovers M Riley RD Simmonds M Stewart G. Preferred reporting items for systematic review and meta-analyses of individual participant data: The PRISMA-IPD statement. JAMA. 2015;313:1657–1665. 10.1001/jama.2015.3656 25919529

[18] Hayden JA van der Windt DA Cartwright JL Côté P Bombardier C. Assessing bias in studies of prognostic factors. Ann Intern Med. 2013;158:280–286. 10.7326/0003-4819-158-4-201302190-00009 23420236

[19] Kleinbaum DG Klein M. Survival Analysis: A Self-Learning Text. Springer; 2012.

[20] Grambsch PM Therneau TM. Proportional hazards tests and diagnostics based on weighted residuals. Biometrika. 1994;81:515–526.

[21] Villa E Cammà C Marietta M Luongo M Critelli R Colopi S. Enoxaparin prevents portal vein thrombosis and liver decompensation in patients with advanced cirrhosis. Gastroenterology. 2012;143:1253–1260.e4. 10.1053/j.gastro.2012.07.018 22819864

[22] de Franchis R Bosch J Garcia-Tsao G Reiberger T Ripoll C Baveno VII Faculty. Baveno VII—Renewing consensus in portal hypertension. J Hepatol. 2022;76:959–974. 10.1016/j.jhep.2021.12.022 PMC11090185 35120736

