# Supplementary material for: Predictors and prognostic impact of spontaneous portal vein thrombosis recanalization in cirrhosis: An individual patient data meta-analysis
Source: Hepatol Commun. 2026 Nov 11;10(12):e01070. doi: 10.1097/HC9.0000000000001070 (PMC13766568; doi:10.1097/HC9.0000000000001070)
Supplement: Supplementary file 1 [file hc9-10-e01070-s001.docx]

**Supplementary material**

**Predictors and Prognostic Impact of Spontaneous Portal Vein Thrombosis Recanalization in Cirrhosis: an Individual Patient Data Meta-analysis**

**Authors:**

Antonio Guerrero* (1, 2) [antonio.guerrero90@hotmail.com](mailto:antonio.guerrero90@hotmail.com)

Laura del Campo* (3) [ldelcamp2@gmail.com](mailto:ldelcamp2@gmail.com)

Angelo Luca (4) [aluca@ismett.edu](mailto:aluca@ismett.edu)

Pierre-Emmanuel Rautou (5,6) [pierre-emmanuel.rautou@inserm.fr](mailto:pierre-emmanuel.rautou@inserm.fr)

Sylvie Chevret (7) sylvie.chevret@u-paris.fr

Leonard Naymagon (8) [leonard.naymagon@mountsinai.org](mailto:leonard.naymagon@mountsinai.org)

Fabio Piscaglia (9,10) [fabio.piscaglia@unibo.it](mailto:fabio.piscaglia@unibo.it)

Horia Ștefănescu (11) horia.stefanescu@irgh.ro

Thomas Reiberger (12) thomas.reiberger@meduniwien.ac.at

Guohong Han (13,14) [hangh@fmmu.edu.cn](mailto:hangh@fmmu.edu.cn)

Francesco Violi (15) [francesco.violi@uniroma1.it](mailto:francesco.violi@uniroma1.it)

Carlos-Noronha Ferreira (16) [carlosnferreira@hotmail.com](mailto:carlosnferreira@hotmail.com)

Luis Téllez (1,2) [luistevilla@gmail.com](mailto:luistevilla@gmail.com)

Bernhard Scheiner (12) bernhard.scheiner@meduniwien.ac.at

Stefania Basili (15) [stefania.basili@uniroma1.it](mailto:stefania.basili@uniroma1.it)

Javier Zamora^†^ (3) [javierza@gmail.com](mailto:javierza@gmail.com)

Agustín Albillos^†^ (1, 2) [agustin.albillos@uah.es](mailto:agustin.albillos@uah.es)

* Share first authorship. ^†^ Share senior authorship

1) Servicio de Gastroenterología y Hepatología, Hospital Universitario Ramón y Cajal, Universidad de Alcalá, Instituto Ramón y Cajal de Investigación Sanitaria (IRYCIS), Madrid, España.

2) Centro de Investigación Biomédica en Red de Enfermedades Hepáticas y Digestivas (CIBEREHD), Instituto Salud Carlos III, Madrid, España.

3) Unidad de Bioestadística Clínica. Hospital Universitario Ramón y Cajal. Instituto Ramón y Cajal de Investigación Sanitaria (IRYCIS), Consorcio de Investigación Biomédica en Red de Epidemiología y Salud Pública (CIBERESP), Madrid, España.

4) Department of Diagnostic and Therapeutic Services Associate Professor of Radiology – University of Pittsburgh. IRCCS-ISMETT, Palermo, Italy.

5) Université Paris-Cité, Inserm, Centre de recherche sur l'inflammation, UMR 1149, Paris, France.

6) AP-HP, Hôpital Beaujon, Service d'Hépatologie, DMU DIGEST, Centre de Référence des Maladies Vasculaires du Foie, FILFOIE, ERN RARE-LIVER, Clichy, France.

7) Institut de Recherche Saint-Louis, équipe ECSTRRA, Université Paris Cité, Paris, France.

8) Tisch Cancer Institute, Icahn School of Medicine at Mount Sinai, New York, NY, United States of America.

9) Division of Internal Medicine, Hepatobiliary and Immunoallergic Diseases, IRCCS Azienda Ospedaliero-Universitaria di Bologna, Italy.

10) Department of Medical and Surgical Sciences, University of Bologna, Italy.

11) Gastroenterology Department, Regional Institute of Gastroenterology and Hepatology O. Fodor, Cluj-Napoca, Romania.

12) Division of Gastroenterology and Hepatology, Department of Medicine III, Medical University of Vienna. Rare Liver Disease (RALID) Center of the European Reference Network for Rare Hepatological Diseases (ERN RARE-LIVER), Medical University Vienna, Vienna, Austria.

13) Department of Liver Diseases and Interventional Radiology, Digestive Diseases Hospital, Xi’an International Medical Center Hospital, Northwestern University, Xi’an, China.

14) Department of Liver Diseases and Digestive Interventional Radiology, National Clinical Research Center for Digestive Diseases and Xijing Hospital of Digestive Diseases, Fourth Military Medical University, Xi’an, China.

15) Department of Internal Medicine, Anestesiology and Cardiovascular Sciences, Sapienza University, Roma, Italy.

16) Servico de Gastrenterologia e Hepatologia, Hospital de Santa Maria, Centro Hospitalar Universitário Lisboa Norte, Clinica Universitaria de Gastrenterologia, Facultad de Medicina, Lisbon, Portugal.

**Suppl. Table 1. Search strategy**

| Database | Platform | Date searched | Number of results |
| --- | --- | --- | --- |
| Medline | Ovid | 08-03-2023 | 58 |
| Embase | Elsevier | 08-03-2023 | 160 |
| CENTRAL-Cochrane Library | Wiley | 08-03-2023 | 5 |
| Total with duplicates |  |  | 223 |
| Total without duplicates |  |  | 183 |

Ovid MEDLINE(R) ALL <1946 to March 07, 2023>

1	Portal Vein/	23423

2	Venous Thrombosis/	29169

3	1 and 2	2517

4	(portal adj4 thrombos#s).ab,ti.	6400

5	3 or 4	7010

6	exp Liver Cirrhosis/	100813

7	cirrhos#s.ab,ti.	102394

8	6 or 7	145729

9	5 and 8	1886

10	Remission, Spontaneous/	16951

11	((natural or spontaneous*) adj6 (remission or recanaliz* or resolution or improve* or reversible or evolution or repermeabilization or regression)).ab,ti.	31128

12	(transient or untreat* or "natural history" or "natural course").ab,ti.	564247

13	10 or 11 or 12	600137

14	9 and 13	116

15	Randomized Controlled Trials as Topic/	160827

16	randomized controlled trial/	588516

17	Random Allocation/	106905

18	Double-Blind Method/	174566

19	Single-Blind Method/	32551

20	Clinical Trial/	537302

21	clinical trial, phase i.pt.	24682

22	clinical trial, phase ii.pt.	39381

23	clinical trial, phase iii.pt.	21486

24	clinical trial, phase iv.pt.	2392

25	controlled clinical trial.pt.	95214

26	randomized controlled trial.pt.	588516

27	multicenter study.pt.	331465

28	clinical trial.pt.	537302

29	exp Clinical Trials as topic/	380865

30	(clinical adj trial$).tw.	466399

31	((singl$ or doubl$ or treb$ or tripl$) adj (blind$3 or mask$3)).tw.	195262

32	Placebos/	35925

33	placebo$.tw.	244050

34	randomly allocated.tw.	35616

35	(allocated adj2 random$).tw.	39334

36	or/15-35	1889731

37	case report.tw.	387139

38	letter/	1209857

39	historical article/	369111

40	or/37-39	1947450

41	36 not 40	1847526

42	Epidemiologic Studies/	9274

43	exp Case-Control Studies/	1397724

44	exp Cohort Studies/	2456525

45	Case control.tw.	150986

46	(cohort adj (study or studies)).tw.	303744

47	Cohort analy$.tw.	11371

48	(Follow up adj (study or studies)).tw.	55569

49	(observational adj (study or studies)).tw.	155079

50	Longitudinal.tw.	313742

51	Retrospective.tw.	720623

52	Cross sectional.tw.	494730

53	Cross-sectional studies/	458978

54	or/42-53	3717266

55	41 or 54	5071797

56	14 and 55	58

Embase (Elsevier)

#65 #14 AND #63 AND ([embase]/lim OR [preprint]/lim)

#64 #14 AND #63 164

#63 #45 OR #62 5,457,095

#62 #46 OR #47 OR #48 OR #49 OR #50 OR #53 OR #54 OR #55 OR #56 OR #57 OR #58 OR #59 OR #60 OR #61 3,717,186

#61 ('cross sectional' NEAR/1 (study OR studies)):ab,ti 322,043

#60 (epidemiologic* NEAR/1 (study OR studies)):ab,ti 121,939

#59 (observational NEAR/1 (study OR studies)):ab,ti 239,833

#58 ('follow up' NEAR/1 (study OR studies)):ab,ti 77,845

#57 (cohort NEAR/1 (study OR studies)):ab,ti 463,867

#56 ('casecontrol' NEAR/1 (study OR studies)):ab,ti 3,017

#55 (cohort NEAR/1 (study OR studies)):ab,ti 463,867

#54 'cohort analysis'/de 963,631

#53 #51 NOT #52824,540

#52 'randomized controlled trial (topic)'/de 246,924

#51 'prospective study'/de 834,518

#50 'retrospective study'/de 1,394,616

#49 'longitudinal study'/de 187,049

#48 'family study'/de 26,274

#47 'case control study'/de 198,386

#46 'clinical study'/de 162,198

#45 #43 NOT #44 2,790,906

#44 #34 OR #35 OR #36 OR #37 OR #38 OR #39 OR #40 OR #41 OR #42 1,890,029

#43 #15 OR #16 OR #17 OR #18 OR #19 OR #20 OR #21 OR #22 OR #23 OR #24 OR #25 OR #26 OR #27 OR #28 OR #29 OR #30 OR #31 OR #32 OR #33 2,857,821

#42 'note':pt 0

#41 'letter':pt 0

#40 'editorial':pt 0

#39 'conference abstract':pt 0

#38 'conference proceeding':pt 4,305

#37 'letter'/de 1,186,897

#36 'abstract report'/de 89,847

#35 'case report':ab,ti 531,833

#34 'case study'/de 91,582

#33 'prospective study'/de 834,518

#32 placebo$:ab,ti 357,452

#31 ((treble OR triple) NEAR/1 blind*):ab,ti 1,886

#30 'double blind*':ab,ti 240,808

#29 'single blind*':ab,ti 30,596

#28 (random* NEAR/2 allocat*):ab,ti 52,890

#27 rct:ab,ti 51,615

#26 "randomi?ed controlled trial$":ab,ti 308,150

#25 'placebo'/de 400,783

#24 'crossover procedure'/de 73,152

#23 'double blind procedure'/de 204,856

#22 'single blind procedure'/de 49,607

#21 'randomization'/exp 96,689

#20 'phase 4 clinical trial'/de 5,193

#19 'phase 3 clinical trial'/de 65,991

#18 'multicenter study'/de 351,754

#17 'controlled clinical trial'/de 439,348

#16 'randomized controlled trial'/de 754,621

#15 'clinical trial'/de 1,069,606

#14 #9 AND #13 361

#13 #10 OR #11 OR #12 1,011,288

#12 transient:ab,ti OR untreat*:ab,ti OR 'natural history':ab,ti OR 'natural course':ab,ti 743,099

#11 ((natural OR spontaneous*) NEAR/6 (remission OR recanaliz* OR resolution OR improve* OR reversible OR evolution OR repermeabilization OR regression)):ab,ti 40,708

#10 'remission'/de 250,085

#9 #5 AND #8 4,200

#8 #6 OR #7 189,967

#7 cirrhos?s:ab,ti 136,083

#6 'liver cirrhosis'/exp 165,027

#5 #3 OR #4 10,336

#4 (portal NEAR/4 thrombos?s):ab,ti 9,333

#3 #1 AND #2 2,288

#2 'vein thrombosis'/exp 131,108

#1 'hepatic portal vein'/exp 31,727

CENTRAL (Cochrane Library)

ID	Search	Hits

#1	MeSH descriptor: [Portal Vein] explode all trees	361

#2	MeSH descriptor: [Venous Thrombosis] explode all trees	3215

#3	#1 AND #2	49

#4	(portal NEAR/3 thrombos?s):ti,ab,kw (Word variations have been searched)	469

#5	#3 OR #4	478

#6	MeSH descriptor: [Liver Cirrhosis] 1 tree(s) exploded	3501

#7	(cirrhos?s):ti,ab,kw (Word variations have been searched)	10926

#8	#6 OR #7	10926

#9	#5 AND #8	173

#10	MeSH descriptor: [Remission, Spontaneous] this term only	581

#11	(((natural or spontaneous*) NEAR/6 (remission or recanaliz* or resolution or improve* or reversible or evolution or repermeabilization or regression))):ti,ab,kw	2305

#12	((transient or untreat* or "natural history" or "natural course")):ti,ab,kw	41417

#13	{OR #10-#12}	43421

#14	#9 AND #13 in Trials	5

**Supplementary Table 2.1. QUIPS risk-of-bias assessment for overall survival in the eight studies included in the meta-analysis.**

| **Domain** | **Luca**  **2012 (7)** | **Nery**  **2015 (3)** | **Violi,**  **2016 (12)** | **Chen,**  **2016 (11)** | **Pettinari,**  **2018 (10)** | **Scheine, 2018 (13)** | **Noronha- Ferreira,**  **2019 (9)** | **Naymagon 2020 (14)** |
| --- | --- | --- | --- | --- | --- | --- | --- | --- |
| Bias due to participation | Low | Low | Low | Low | Low | Low | Low | Low |
| Bias in study attrition | Moderate | Moderate | Moderate | Moderate | Moderate | High | Moderate | Moderate |
| Bias in prognostic factor measurement | Low | Low | Low | Low | Low | Moderate | Low | Low |
| Bias due to outcome measurement | Low | Low | Low | Low | Low | Low | Low | Low |
| Bias due to study confounding | Low | low | Low | Low | Low | Low | Low | Low |
| Bias in statistical analysis and reported result | Low | Low | Low | Low | Low | Low | Low | Low |
| **Overall**  **bias** | Moderate | Moderate | Moderate | Moderate | Moderate | High | Moderate | Moderate |

**Supplementary Table 2.2. QUIPS risk-of-bias assessment for spontaneous recanalization in the eight studies included in the meta-analysis.**

| **Domain** | **Luca 2012**  **(7)** | **Nery 2015**  **(3)** | **Violi, 2016 (12)** | **Chen,**  **2016**  **(11)** | **Pettinari,**  **2018**  **(10)** | **Scheiner, 2018**  **(13)** | **Noronha- Ferreira,**  **2019 (9)** | **Naymagon 2020**  **(14)** |
| --- | --- | --- | --- | --- | --- | --- | --- | --- |
| Bias due to participation | Low | Low | Low | Low | Low | Low | Low | Low |
| Bias in study attrition | Moderate | Moderate | Moderate | Moderate | Moderate | High | Moderate | Moderate |
| Bias in prognostic factor measurement | Low | Low | Low | Low | Low | Moderate | Low | Low |
| Bias due to outcome measurement | Moderate | Moderate | Moderate | Low | Moderate | High | Low | Low |
| Bias due to study confounding | Low | Low | Low | Low | Low | Low | Low | Low |
| Bias in statistical analysis and reported result | Low | Low | Low | Low | Low | Low | Low | Low |
| **Overall**  **bias** | Moderate | Moderate | Moderate | Moderate | Moderate | High | Moderate | Moderate |

**Supplementary Tables 3–10. Patient characteristics by individual study.**

| Luca et al |  |  |
| --- | --- | --- |
|  | N = 42 | Missings (%) |
| age, mean (SD) | 56.2 (7.4) | 0 (0.0%) |
| Min - max | 40 - 65 |  |
| sex |  | 0 (0.0%) |
| Male | 29 (69.0%) |  |
| Female | 13 (31.0%) |  |
| etiology |  | 0 (0.0%) |
| Viral | 33 (78.6%) |  |
| Alcohol | 1 (2.4%) |  |
| Other | 8 (19.0%) |  |
| Child-Pugh |  |  |
| Mean (SD) | 8.1 (1.9) | 0 (0.0%) |
| Child-Pugh A | 8 (19.0%) | 0 (0.0%) |
| Child-Pugh B/C | 34 (81.0%) | 0 (0.0%) |
| MELD score |  |  |
| Mean (SD) | 12.3 (2.9) | 0 (0.0%) |
| Betablock | 19 (45.2%) | 0 (0.0%) |
| varices | 37 (88.1%) | 0 (0.0%) |
| Platelets, Median [p25-p75] | 57500.0 (48000.0-85000.0) | 0 (0.0%) |
| Min - max | 21000 - 147000 |  |
| Extent of PVT |  | 0 (0.0%) |
| Partial | 42 (100.0%) |  |
| Complete | 0 |  |
| localization |  | 0 (0.0%) |
| Main trunk | 42 (100.0%) |  |
| Intrahepatic | 0 |  |
| Mesenteric | 0 |  |
| status_trombo |  | 0 (0.0%) |
| No recanalization | 23 (54.8%) |  |
| Partial recanalization | 18 (42.9%) |  |
| Total recanalization | 1 (2.4%) |  |
| status_fu |  | 0 (0.0%) |
| Alive | 22 (52.4%) |  |
| Death | 12 (28.6%) |  |
| Liver transplantation | 8 (19.0%) |  |

| Nery et al |  |  |
| --- | --- | --- |
|  | N = 118 | Missings (%) |
| age, mean (SD) | 55.9 (9.7) | 1 (0.8%) |
| Min - max | 29 - 74 |  |
| sex |  | 1 (0.8%) |
| Male | 82 (70.1%) |  |
| Female | 35 (29.9%) |  |
| etiology |  | 0 (0.0%) |
| Viral | 55 (46.6%) |  |
| Alcohol | 55 (46.6%) |  |
| Other | 8 (6.8%) |  |
| Child-Pugh |  |  |
| Mean (SD) | 1.2 (0.4) | 12 (10.2%) |
| Child-Pugh A | 88 (83.0%) | 12 (10.2%) |
| Child-Pugh B/C | 18 (17.0%) | 12 (10.2%) |
| MELD score |  |  |
| Mean (SD) | 10.0 (2.3) | 34 (28.8%) |
| Beta-blockers | 2 (1.7%) | 0 (0.0%) |
| varices | 72 (73.5%) | 20 (16.9%) |
| Platelets, Median [p25-p75] | 114000.0 (78000.0-161000.0) | 56 (47.5%) |
| Min - max | 9000 - 317000 |  |
| Extent of PVT |  | 0 (0.0%) |
| Partial | 96 (81.4%) |  |
| Complete | 22 (18.6%) |  |
| localization |  | 2 (1.7%) |
| Main trunk | 89 (76.7%) |  |
| Intrahepatic | 27 (23.3%) |  |
| Mesenteric | 0 |  |
| status_trombo |  | 19 (16.1%) |
| No recanalization | 4 (4.0%) |  |
| Partial recanalization | 2 (2.0%) |  |
| Total recanalization | 93 (93.9%) |  |
| status_fu |  | 0 (0.0%) |
| Alive | 101 (85.6%) |  |
| Death | 13 (11.0%) |  |
| Liver transplantation | 4 (3.4%) |  |

| Violi et al |  |  |
| --- | --- | --- |
|  | N = 82 | Missings (%) |
| age, mean (SD) | 67.4 (11.4) | 0 (0.0%) |
| Min - max | 22 - 87 |  |
| sex |  | 0 (0.0%) |
| Male | 24 (29.3%) |  |
| Female | 58 (70.7%) |  |
| etiology |  | 0 (0.0%) |
| Viral | 45 (54.9%) |  |
| Alcohol | 33 (40.2%) |  |
| Other | 4 (4.9%) |  |
| Child-Pugh |  |  |
| Mean (SD) | 7.6 (2.1) | 0 (0.0%) |
| Child-Pugh A | 31 (37.8%) | 0 (0.0%) |
| Child-Pugh B/C | 51 (62.2%) | 0 (0.0%) |
| MELD score |  |  |
| Mean (SD) | 12.8 (5.0) | 1 (1.2%) |
| Beta-blockers | 47 (57.3%) | 0 (0.0%) |
| varices | 60 (74.1%) | 1 (1.2%) |
| Platelets, Median [p25-p75] | 91000.0 (66000.0-127000.0) | 5 (6.1%) |
| Min - max | 28000 - 308000 |  |
| Extent of PVT |  | 1 (1.2%) |
| Partial | 62 (76.5%) |  |
| Complete | 19 (23.5%) |  |
| localization |  | 0 (0.0%) |
| Main trunk | 55 (67.1%) |  |
| Intrahepatic | 24 (29.3%) |  |
| Mesenteric | 3 (3.7%) |  |
| status_trombo |  | 14 (17.1%) |
| No recanalization | 15 (22.1%) |  |
| Partial recanalization | 1 (1.5%) |  |
| Total recanalization | 52 (76.5%) |  |
| status_fu |  | 0 (0.0%) |
| Alive | 56 (68.3%) |  |
| Death | 26 (31.7%) |  |
| Liver transplantation | 0 |  |
|  |  |  |

| Chen et al |  |  |
| --- | --- | --- |
|  | N = 29 | Missings (%) |
| age, mean (SD) | 46.3 (11.0) | 0 (0.0%) |
| Min - max | 29 - 78 |  |
| sex |  | 0 (0.0%) |
| Male | 21 (72.4%) |  |
| Female | 8 (27.6%) |  |
| etiology |  | 0 (0.0%) |
| Viral | 18 (62.1%) |  |
| Alcohol | 1 (3.4%) |  |
| Other | 10 (34.5%) |  |
| Child-Pugh |  |  |
| Mean (SD) | 6.7 (1.9) | 0 (0.0%) |
| Child-Pugh A | 17 (58.6%) | 0 (0.0%) |
| Child-Pugh B/C | 12 (41.4%) | 0 (0.0%) |
| MELD score |  |  |
| Mean (SD) | 9.7 (2.7) | 0 (0.0%) |
| Beta-blockers | 1 (3.4%) | 0 (0.0%) |
| varices | 23 (79.3%) | 0 (0.0%) |
| Platelets, Median [p25-p75] | 143000.0 (95000.0-217000.0) | 0 (0.0%) |
| Min - max | 25000 - 390000 |  |
| Extent of PVT |  | 0 (0.0%) |
| Partial | 10 (34.5%) |  |
| Complete | 19 (65.5%) |  |
| localization |  | 0 (0.0%) |
| Main trunk | 29 (100.0%) |  |
| Intrahepatic | 0 |  |
| Mesenteric | 0 |  |
| status_trombo |  | 0 (0.0%) |
| No recanalization | 25 (86.2%) |  |
| Partial recanalization | 3 (10.3%) |  |
| Total recanalization | 1 (3.4%) |  |
| status_fu |  | 0 (0.0%) |
| Alive | 23 (79.3%) |  |
| Death | 6 (20.7%) |  |
| Liver transplantation | 0 |  |

| Pettinari et al |  |  |
| --- | --- | --- |
|  | N = 104 | Missings (%) |
| age, mean (SD) | 57.8 (11.4) | 0 (0.0%) |
| Min - max | 7 - 84 |  |
| sex |  | 0 (0.0%) |
| Male | 75 (72.1%) |  |
| Female | 29 (27.9%) |  |
| etiology |  | 1 (1.0%) |
| Viral | 54 (52.4%) |  |
| Alcohol | 34 (33.0%) |  |
| Other | 15 (14.6%) |  |
| Child-Pugh |  |  |
| Mean (SD) | 7.5 (2.0) | 0 (0.0%) |
| Child-Pugh A | 39 (37.5%) | 0 (0.0%) |
| Child-Pugh B/C | 65 (62.5%) | 0 (0.0%) |
| MELD score |  |  |
| Mean (SD) | 14.3 (4.2) | 4 (3.8%) |
| Beta-blockers | 80 (77.7%) | 1 (1.0%) |
| varices | 96 (92.3%) | 0 (0.0%) |
| Platelets, Median [p25-p75] | 70500.0 (54000.0-108000.0) | 14 (13.5%) |
| Min - max | 19000 - 449000 |  |
| Extent of PVT |  | 4 (3.8%) |
| Partial | 86 (86.0%) |  |
| Complete | 14 (14.0%) |  |
| localization |  | 4 (3.8%) |
| Main trunk | 75 (75.0%) |  |
| Intrahepatic | 20 (20.0%) |  |
| Mesenteric | 5 (5.0%) |  |
| status_trombo |  | 0 (0.0%) |
| No recanalization | 78 (75.0%) |  |
| Partial recanalization | 13 (12.5%) |  |
| Total recanalization | 13 (12.5%) |  |
| status_fu |  | 0 (0.0%) |
| Alive | 67 (64.4%) |  |
| Death | 18 (17.3%) |  |
| Liver transplantation | 19 (18.3%) |  |

| Scheiner et al |  |  |
| --- | --- | --- |
|  | N = 30 | Missings (%) |
| age, mean (SD) | 54.3 (11.6) | 0 (0.0%) |
| Min - max | 32 - 72 |  |
| sex |  | 0 (0.0%) |
| Male | 24 (80.0%) |  |
| Female | 6 (20.0%) |  |
| etiology |  | 0 (0.0%) |
| Viral | 5 (16.7%) |  |
| Alcohol | 17 (56.7%) |  |
| Other | 8 (26.7%) |  |
| Child-Pugh |  |  |
| Mean (SD) | 8.7 (2.7) | 0 (0.0%) |
| Child-Pugh A | 8 (26.7%) | 0 (0.0%) |
| Child-Pugh B/C | 22 (73.3%) | 0 (0.0%) |
| MELD score |  |  |
| Mean (SD) | 14.0 (6.8) | 0 (0.0%) |
| Beta-blockers | 16 (53.3%) | 0 (0.0%) |
| varices | 22 (73.3%) | 0 (0.0%) |
| Platelets, Median [p25-p75] | 96500.0 (53000.0-201000.0) | 0 (0.0%) |
| Min - max | 25000 - 561000 |  |
| Extent of PVT |  | 0 (0.0%) |
| Partial | 18 (60.0%) |  |
| Complete | 12 (40.0%) |  |
| localization |  | 0 (0.0%) |
| Main trunk | 30 (100.0%) |  |
| Intrahepatic | 0 |  |
| Mesenteric | 0 |  |
| status_trombo |  | 0 (0.0%) |
| No recanalization | 22 (73.3%) |  |
| Partial recanalization | 1 (3.3%) |  |
| Total recanalization | 7 (23.3%) |  |
| status_fu |  | 0 (0.0%) |
| Alive | 13 (43.3%) |  |
| Death | 14 (46.7%) |  |
| Liver transplantation | 3 (10.0%) |  |

| Noronha-Ferreira et al |  |  |
| --- | --- | --- |
|  | N = 54 | Missings (%) |
| age, mean (SD) | 58.7 (12.2) | 0 (0.0%) |
| Min - max | 20 - 77 |  |
| sex |  | 0 (0.0%) |
| Male | 33 (61.1%) |  |
| Female | 21 (38.9%) |  |
| etiology |  | 0 (0.0%) |
| Viral | 13 (24.1%) |  |
| Alcohol | 31 (57.4%) |  |
| Other | 10 (18.5%) |  |
| Child-Pugh |  |  |
| Mean (SD) | 8.7 (2.4) | 0 (0.0%) |
| Child-Pugh A | 11 (20.4%) | 0 (0.0%) |
| Child-Pugh B/C | 43 (79.6%) | 0 (0.0%) |
| MELD score |  |  |
| Mean (SD) | 14.0 (5.9) | 1 (1.9%) |
| Betablock | 7 (35.0%) | 34 (63.0%) |
| varices | 38 (92.7%) | 13 (24.1%) |
| Platelets, Median [p25-p75] | 85000.0 (54000.0-143000.0) | 2 (3.7%) |
| Min - max | 11000 - 528000 |  |
| Extent of PVT |  | 6 (11.1%) |
| Partial | 26 (54.2%) |  |
| Complete | 22 (45.8%) |  |
| localization |  | 0 (0.0%) |
| Main trunk | 50 (92.6%) |  |
| Intrahepatic | 4 (7.4%) |  |
| Mesenteric | 0 |  |
| status_trombo |  | 0 (0.0%) |
| No recanalization | 46 (85.2%) |  |
| Partial recanalization | 4 (7.4%) |  |
| Total recanalization | 4 (7.4%) |  |
| status_fu |  | 1 (1.9%) |
| Alive | 15 (28.3%) |  |
| Death | 35 (66.0%) |  |
| Liver transplantation | 3 (5.7%) |  |

| Naymagon |  |  |
| --- | --- | --- |
|  | N = 127 | Missings (%) |
| age, mean (SD) | 59.4 (10.6) | 0 (0.0%) |
| Min - max | 29 - 83 |  |
| sex |  | 0 (0.0%) |
| Male | 90 (70.9%) |  |
| Female | 37 (29.1%) |  |
| etiology |  | 0 (0.0%) |
| Viral | 51 (40.2%) |  |
| Alcohol | 26 (20.5%) |  |
| Other | 50 (39.4%) |  |
| Child-Pugh |  |  |
| Mean (SD) | 8.5 (2.0) | 0 (0.0%) |
| Child-Pugh A | 19 (15.0%) | 0 (0.0%) |
| Child-Pugh B/C | 108 (85.0%) | 0 (0.0%) |
| MELD score |  |  |
| Mean (SD) | 13.5 (5.7) | 0 (0.0%) |
| Betablock | 79 (62.2%) | 0 (0.0%) |
| varices | 105 (82.7%) | 0 (0.0%) |
| Platelets, Median [p25-p75] | 82000.0 (55000.0-135000.0) | 0 (0.0%) |
| Min - max | 19000 - 337000 |  |
| Extent of PVT |  | 0 (0.0%) |
| Partial | 98 (77.2%) |  |
| Complete | 29 (22.8%) |  |
| localization |  | 3 (2.4%) |
| Main trunk | 88 (71.0%) |  |
| Intrahepatic | 30 (24.2%) |  |
| Mesenteric | 6 (4.8%) |  |
| status_trombo |  | 0 (0.0%) |
| No recanalization | 76 (59.8%) |  |
| Partial recanalization | 17 (13.4%) |  |
| Total recanalization | 34 (26.8%) |  |
| status_fu |  | 0 (0.0%) |
| Alive | 79 (62.2%) |  |
| Death | 38 (29.9%) |  |
| Liver transplantation | 10 (7.9%) |  |

**Supplementary Table 11. Adjusted sub-hazard ratios (aSHR) from the final multivariable competing-risk model assessing the effect of spontaneous recanalization on all-cause mortality.**

| **Variable** | **aSHR [95% conf. interval]** | **p-value** |
| --- | --- | --- |
| **Spontaneous recanalization** | **0.51 [0.28; 0.91]** | **0.024** |
| **Age**, (years) | **1.04 [1.03; 1.06]** | **<0.001** |
| **MELD**, points | **1.10 [1.05; 1.13]** | **<0.001** |
| **Child-Pugh** |  |  |
| A | [reference] |  |
| B/C | **2.2 [1.50; 3.24]** | **<0.001** |
| **Beta-blockers** | **0.65 [0.51; 0.83]** | **0.001** |
| **Etiology** |  |  |
| Viral | [reference] |  |
| Alcohol | 1.01 [0.53; 1.92] | 0.967 |
| Others | 0.88 [0.56; 1.39] | 0.599 |
| **Portal vein thrombosis severity** |  |  |
| Partial | [reference] |  |
| Complete | 0.88 [0.66; 1.17] | 0.38 |
| **Portal vein thrombosis localization** |  |  |
| Main trunk | [reference] |  |
| Intrahepatic | 0.92 [0.52; 1.60] | 0.765 |
| Mesenteric | 0.81 [0.44; 1.51] | 0.512 |
| **Platelets**, 10^3^/µL | 0.94 [0.60; 1.49] | 0.808 |
| **Varices** | 0.70 [0.36; 1.36] | 0.295 |

**Supplementary Table 12. Sensitivity analysis: Adjusted sub-hazard ratios (aSHR) from the final multivariable competing-risks model assessing the association of spontaneous recanalization on all-cause mortality in the overall cohort and in the cohort patients with thrombosis of the main portal vein trunk**

| **Variable** | **Category** | **Overall cohort**  aSHR  [95% conf. interval] | **Main portal vein trunk thrombosis**  aSHR  [95% conf. interval] |
| --- | --- | --- | --- |
| **Recanalization status (ref: no recanalization)** | | | |
| Thrombosis status | Partial recanalization | 0.67 (0.32-1.40), p=0.286 | 0.68 (0.31-1.52), p=0.348 |
| Thrombosis status | Complete recanalization | 0.45 (0.21-0.95), p=0.036 | 0.38 (0.16-0.90), p=0.027 |
| **Covariates** | | | |
| Age (years) |  | 1.04 (1.03-1.06), p<0.001 | 1.04 (1.03-1.06), p<0.001 |
| Sex (female) |  | 1.31 (0.78-2.19), p=0.309 | 1.61 (1.05-2.48), p=0.030 |
| MELD |  | 1.10 (1.06-1.14), p<0.001 | 1.13 (1.08-1.19), p<0.001 |
| Child-Pugh |  | 2.09 (1.47-2.97), p<0.001 | 1.73 (1.05-2.86), p=0.031 |
| Beta-blockers |  | 0.65 (0.52-0.81), p<0.001 | 0.69 (0.52-0.93), p=0.014 |
| Etiology (ref: viral) | Alcohol | 1.05 (0.51-2.13), p=0.900 | 0.96 (0.47-1.95), p=0.908 |
| Etiology (ref: viral) | Other | 0.89 (0.55-1.44), p=0.621 | 0.80 (0.49-1.29), p=0.359 |
| Degree of thrombosis |  | 0.88 (0.66-1.17), p=0.388 | 0.88 (0.51-1.52), p=0.638 |
| Localization (ref: main trunk) | Intrahepatic | 0.95 (0.57-1.59), p=0.854 | — (excluded by design) |
| Localization (ref: main trunk) | Mesenteric | 0.84 (0.43-1.65), p=0.618 | — (excluded by design) |
| log-Platelets |  | 0.95 (0.60-1.51), p=0.831 | 1.16 (0.74-1.80), p=0.520 |
| Varices |  | 0.70 (0.36-1.36), p=0.292 | 0.82 (0.38-1.75), p=0.610 |

**Supplementary Table 13. Sensitivity analysis: Adjusted sub-hazard ratios (aSHR) from the final multivariable competing-risks model assessing the association of any (partial or complete) spontaneous recanalization on all-cause mortality in the overall cohort and in the cohort of patients with thrombosis of the main portal vein trunk**

| **Variable** | **Category** | **Overall cohort**  aSHR  [95% conf. interval] | **Main portal vein trunk thrombosis**  aSHR  [95% conf. interval] |
| --- | --- | --- | --- |
| Thrombosis status | Any recanalization vs. none | 0.51 (0.29-0.92), p=0.024 | 0.48 (0.24-0.99), p=0.046 |
| **Covariates** | | | |
| Age (years) |  | 1.04 (1.03-1.06), p<0.001 | 1.04 (1.02-1.05), p<0.001 |
| Sex (female) |  | 1.28 (0.76-2.17), p=0.354 | 1.57 (0.99-2.50), p=0.056 |
| MELD |  | 1.10 (1.06-1.14), p<0.001 | 1.12 (1.08-1.17), p<0.001 |
| Child-Pugh |  | 2.20 (1.50-3.24), p<0.001 | 1.88 (1.14-3.12), p=0.014 |
| Beta-blockers |  | 0.65 (0.51-0.83), p=0.001 | 0.70 (0.50-0.97), p=0.032 |
| Etiology  (ref: viral) | Alcohol | 1.01 (0.53-1.93), p=0.967 | 0.91 (0.48-1.74), p=0.776 |
| Etiology  (ref: viral) | Other | 0.88 (0.56-1.40), p=0.599 | 0.80 (0.52-1.24), p=0.324 |
| Degree of thrombosis |  | 0.88 (0.66-1.17), p=0.380 | 0.87 (0.51-1.48), p=0.611 |
| Localization (ref: main trunk) | Intrahepatic | 0.92 (0.52-1.61), p=0.765 | — (excluded by design) |
| Localization (ref: main trunk) | Mesenteric | 0.81 (0.44-1.51), p=0.512 | — (excluded by design) |
| log-Platelets |  | 0.95 (0.60-1.49), p=0.808 | 1.15 (0.75-1.77), p=0.522 |
| Varices |  | 0.70 (0.36-1.36), p=0.295 | 0.85 (0.42-1.73), p=0.653 |

**Supplementary Table 14. Sensitivity analysis: Multivariable ordinal logistic regression analysis of baseline covariates associated with a higher probability of thrombosis recanalization in the overall cohort and in the cohort of patients with thrombosis of the main portal vein trunk**

| **Variable** | **Category** | **Overall cohort**  OR [95% conf. interval] | **Main portal vein trunk thrombosis**  [95% conf. interval] |
| --- | --- | --- | --- |
| Age (years) |  | 1.04 (0.99-1.09), p=0.084 | 1.05 (1.00-1.10), p=0.069 |
| Beta-blockers |  | 0.56 (0.27-1.13), p=0.103 | 0.67 (0.31-1.45), p=0.309 |
| Child-Pugh (ref: B/C) | Child-Pugh A | 2.61 (1.18-5.79), p=0.018 | 2.73 (1.26-5.90), p=0.011 |
| Etiology  (ref: viral) | Alcohol | 0.90 (0.60-1.36), p=0.630 | 0.71 (0.34-1.49), p=0.363 |
| Etiology  (ref: viral) | Other | 0.57 (0.23-1.39), p=0.216 | 0.49 (0.19-1.26), p=0.140 |
| Degree of thrombosis (ref: complete) | Partial | 1.68 (1.09-2.59), p=0.018 | 2.15 (1.06-4.37), p=0.034 |
| Localization (ref: main trunk) | No main trunk (intrahepatic/mesenteric) | 2.03 (1.23-3.33), p=0.005 | — (excluded by design) |
| log-Platelets |  | 0.92 (0.64-1.31), p=0.639 | 0.83 (0.57-1.21), p=0.333 |
| MELD |  | 1.31 (0.86-2.00), p=0.205 | 1.06 (0.59-1.90), p=0.857 |
| Sex (female) |  | 1.19 (0.89-1.59), p=0.238 | 1.21 (0.78-1.87), p=0.396 |
| Varices |  | 1.11 (0.57-2.17), p=0.758 | 0.66 (0.36-1.23), p=0.191 |
